# Advances in the Relationships Between Cow’s Milk Protein Allergy and Gut Microbiota in Infants

**DOI:** 10.3389/fmicb.2021.716667

**Published:** 2021-08-16

**Authors:** Yudie Yang, Xiaoqi Li, Ying Yang, Saeed Shoaie, Cheng Zhang, Boyang Ji, Yongjun Wei

**Affiliations:** ^1^Key Laboratory of Advanced Drug Preparation Technologies, Ministry of Education, School of Pharmaceutical Sciences, Henan Children’s Hospital, Zhengzhou Children’s Hospital, Children’s Hospital Affiliated to Zhengzhou University, Zhengzhou University, Zhengzhou, China; ^2^Jing’an District Central Hospital of Shanghai, Jing’an Branch, Huashan Hospital, Fudan University, Shanghai, China; ^3^Science for Life Laboratory, KTH Royal Institute of Technology, Stockholm, Sweden; ^4^Faculty of Dentistry, Oral and Craniofacial Sciences, Centre for Host-Microbiome Interactions, King’s College London, London, United Kingdom; ^5^Department of Biology and Biological Engineering, Chalmers University of Technology, Gothenburg, Sweden; ^6^Laboratory of Synthetic Biology, Zhengzhou University, Zhengzhou, China

**Keywords:** cow’s milk allergy, gut microbiota, probiotics, prebiotics, synthetic microbiota, fecal microbiota transplantation

## Abstract

Cow’s milk protein allergy (CMPA) is an immune response to cow’s milk proteins, which is one of the most common food allergies in infants and young children. It is estimated that 2–3% of infants and young children have CMPA. The diet, gut microbiota, and their interactions are believed to be involved in the alterations of mucosal immune tolerance, which might lead to the development of CMPA and other food allergies. In this review, the potential molecular mechanisms of CMPA, including omics technologies used for analyzing microbiota, impacts of early microbial exposures on CMPA development, and microbiota–host interactions, are summarized. The probiotics, prebiotics, synbiotics, fecal microbiota transplantation, and other modulation strategies for gut microbiota and the potential application of microbiota-based design of diets for the CMPA treatment are also discussed. This review not only summarizes the current studies about the interactions of CMPA with gut microbiota but also gives insights into the possible CMPA treatment strategies by modulating gut microbiota, which might help in improving the life quality of CMPA patients in the future.

## Introduction

Food allergy has become a major public health issue worldwide. The prevalence of food allergies has been growing steadily, affecting 3–6% of the United States population (Sicherer and Sampson, 2014). Cow’s milk protein allergy (CMPA) is one of the most common food allergies in early childhood, affecting 2–3% of the children under 3 years of age (Sicherer, 2011; Savage and Johns, 2015). Besides, 1% of adults show severe allergic reactions related to milk consumption (Nwaru et al., 2014; Schocker et al., 2019). CMPA is an immune response to cow’s milk proteins derived from the infant formula. The infants suffering from CMPA cannot consume cow’s milk and need amino acid-based formula (AAF) or extensively hydrolyzed casein formula (EHCF). However, some infants are intolerant to AAF and EHCF. Therefore, CMPA decreases the life quality of infants, affects their health, and causes financial burdens to their families (Vanderhoof and Kleinman, 2015). Altogether, the exploration of the molecular mechanisms of CMPA might be a crucial step to developing cost-effective treatment strategies for CMPA.

Based on the immune responses, CMPA can be classified into three types, including the non-immunoglobulin E (IgE)-mediated CMPA, IgE-mediated CMPA, and mixed CMPA. The IgE-mediated CMPA is the most common form of CMPA (Wiley et al., 2015), which often occurs at the first-time exposure of infants to cow’ milk (infant formula or other foods, containing cow’s milk or cereal). It could be diagnosed when the infants have a history of immediate, acute, and objective symptoms within 2 h after the ingestion of dairy products (D’Auria and Venter, 2020; Munblit et al., 2020). Within 2 h of the exposure, the infants with IgE-mediated CMPA experience erythema, angioedema, urticaria, vomiting, lethargy, or respiratory symptoms, which can vanish soon and happen again upon exposure to cow’s milk. In contrast, the non-IgE-mediated CMPA occurs without stable symptoms and no efficient diagnostic methods are available (Flom and Sicherer, 2019). The non-IgE-mediated CMPA appears at least 2 h after exposure to cow’s milk, which is usually accompanied by the food protein-induced enterocolitis syndrome, allergic proctocolitis, chronic cutaneous, or gastrointestinal symptoms. Since the symptoms are similar to the other infant diseases, the delayed diagnosis and misdiagnosis of non-IgE-mediated CMPA are common in clinical practices (Munblit et al., 2020). The mixed CMPA is a combination of the IgE- and non-IgE-mediated CMPA and is sophisticated in diagnosis and treatment. Therefore, the investigation of its molecular mechanisms is necessary for effective treatment.

## Gut Microbiota in Early Childhood

Human bodies are colonized by various microorganisms, the most influential of which is gut microbiota (Sprockett et al., 2018). The wide applications of next-generation sequencing (NGS) technologies have potentiated the investigation of the structure and function of gut microbiota in a cost-effective way (Berni Canani et al., 2019; Liu et al., 2021). The 16S rRNA gene sequencing technology is used to identify the composition of gut microbiota at the genus and phylum levels, while the shotgun metagenomic analysis and other strategies are used to explore the taxonomic and functional compositions of gut microbiota at species or strain level (Supplementary Figure 1; Bunyavanich and Berin, 2019). The differences at the strain-level diversity of microbiota have shown distinct effects on the host phenotypes (Chen et al., 2018). Therefore, the understanding of interspecies diversity is important for the development of microbiome-based biomarkers linked to human health and disease (Bunyavanich and Berin, 2019). The integration of multi-omics technologies, including genomics, epigenomics, transcriptomics, proteomics, metabolomics, and microbiome, can help in the investigation, characterization, and quantification of microorganisms in human gut microbiota, giving insights into the interactions between host and its gut microbiota (Dhondalay et al., 2018; Shi et al., 2020). Based on the functional properties and microbial associations, the potential microbial biomarkers can be identified and the personalized medicine protocols, including drugs, live probiotics, and microbial metabolites, can be designed for treatment purposes (Sicherer and Sampson, 2018; Berni Canani et al., 2019).

The microbiota has close interactions with the homeostasis of immune response and various interconnections with the host metabolic pathways (Conway and Boddy, 2013; Gensollen et al., 2016). The microbial colonization in early life strongly affects humans’ health and diseases for their whole lifetime (Turnbaugh et al., 2009; Benson et al., 2010; Gensollen et al., 2016). The gut microbiota changes dramatically during the first year of life and is relatively stable and mature after 3 years of age (Yatsunenko et al., 2012; Stewart et al., 2018). The maturation of gut microbiome can be divided into the developmental phase (months 3–14), transitional phase (months 15–30), and stable phase (months 31–46) (Stewart et al., 2018). Understanding the colonization process of gut microbiota in early life is critical for further understanding CMPA and other food allergies. The total amounts of gut microbes (especially anaerobic microbes) in children with CMPA are relatively higher as compared to those of healthy children (Bunyavanich and Schadt, 2015). The composition of gut microbiota at the age of 3–6 months was found to be associated with milk allergy by the age of 8 years with the enrichment of class Clostridia and phylum Firmicutes in the infant’s gut microbiota (Bunyavanich et al., 2016). Moreover, a recent study has shown that the newborns, who developed IgE-mediated allergic sensitization by 1 year of their age, exhibited less diverse gut metabolic activities at their birth, and the specific metabolic clusters were associated with the abundance of key taxa, driving the maturation of gut microbiota (Petersen et al., 2021).

The progression of microbiota is associated with various perinatal characteristics, such as mode of delivery, type of feeding, lifestyle, antibiotic usage, and geographic distribution (Adak and Khan, 2019). The mode of delivery affects the initial infant’s gut microbiota (Biasucci et al., 2010; Wampach et al., 2018) and maturation (Chu et al., 2017). The vaginally delivered newborns mainly obtain their gut microbiota from the mother’s vaginal microbiota, and the gut microbiota is mainly composed of the genera *Lactobacillus* and *Prevotella*; on the other hand, the gut microbiota of cesarean section-delivered newborns is similar to the skin microbiota, which is mainly composed of the genera *Staphylococcus*, *Corynebacterium*, and *Propionibacterium* (Dominguez-Bello et al., 2010; Tamburini et al., 2016). After birth, the microorganisms from the mother and the surrounding environments colonize rapidly (Vandenplas et al., 2020) and some opportunistic pathogens might also colonize the infants’ gut, causing infections (Shao et al., 2019). The initial gut microbiota subsequently affects child health, and the cesarean section-delivered newborns might have a higher risk of food allergies and other diseases (Arrieta et al., 2015; Rachid and Chatila, 2016). Moreover, the metabolic maturation of infants’ gut microbiota can be predicted by the feeding types and maternal gestational weight gain (Bäckhed et al., 2015; Baumann-Dudenhoeffer et al., 2018). Further insights into the development of an infant’s gut microbiota suggested that it was affected by diverse environmental factors, including diet, breastfeeding, and antibiotics (Bokulich et al., 2016; Yassour et al., 2016; Bazanella et al., 2017), and the gut microbiota was not mature even at the age of 5 (Roswall et al., 2021).

## Microbiota–Host Interactions and CMPA

**FIGURE 1:**
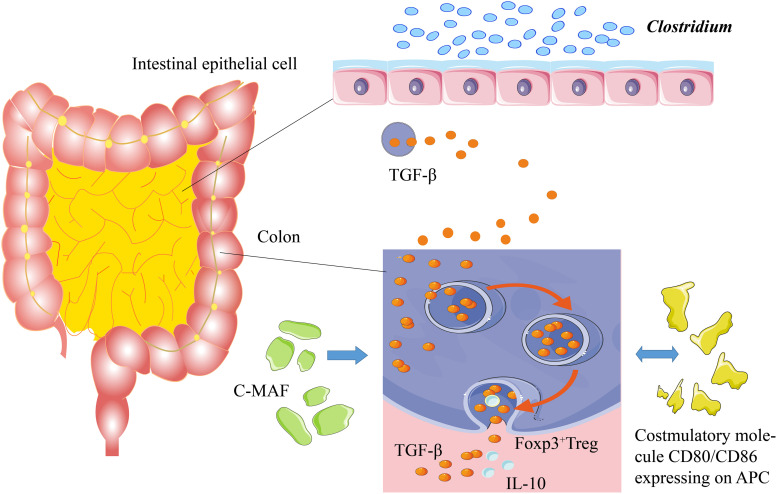
Mechanism of immune tolerance. Some *Clostridium* species stimulate the intestines to secrete TGF-β. TGF-β can induce Foxp3^+^ Treg cells in the colon to secrete IL-10, thereby promoting immune tolerance. c-MAF, a T cell transcription factor, is the key factor to sustain the stability of gut microbiota. The costimulatory molecules of CD80/CD86, which are expressed on the antigen-presenting cells, stimulate the secretion of TGF-β and IL-10.

A healthy immune system is tolerant to self-antigens and only shows allergic reactions to the foreign antigens, such as pathogens (Stephen-Victor and Chatila, 2019). The food-allergic infants are sensitive to the specific food antigens with the assistance of a pathogenic T-helper 2 (Th2) response (Johnston et al., 2014). Dendritic cells (DCs) and specific antigen-presenting cells (APCs) are widely distributed in the human body, playing essential roles in immune responses. Usually, the DCs can identify and process foreign antigens, as well as the injured host cells, thereby inducing juvenile T cells to activate the adaptive immune responses. On the other hand, the DC can limit the response of effector cells by developing central and peripheral tolerance (Qian and Cao, 2018). The gut-draining lymph nodes (gLNs) show distinct immune functions in the different areas of the gut. The gene expression of DC in gLNs is diverse, resulting in different immune responses against the same antigen. The gLNs can determine the host’s adaptive immune responses *via* the compartmentalization of the gut into segments for different antigens (Esterhazy et al., 2019). The DC promotes immune tolerance by inducing and inhibiting T cell response, accelerating apoptosis, and producing regulatory T (Treg) cells (Kushwah and Hu, 2011). Unlike the Treg cells in other organs, the intestinal Treg antigen receptor (TCR) can inhibit the immune responses to harmless antigens and symbiotic microbiota in diet (Tanoue et al., 2016). The regulation of Treg cells in intestinal immunity at a steady state is essential for sustaining tolerogenic response by adaptive immunity. Some species in the *Clostridium* genus or other species can induce the generation of colonic Foxp3^+^ Treg cells by transforming growth factor-beta (TGF-β). Meanwhile, the CD80/86 proteins expressed by the Treg cells can inhibit CD28 of effector T cells by releasing TGF-β and interleukin-10 (IL-10) to mediate the immune tolerance (Figure 1; Russler-Germain et al., 2017).

Intestinal Treg cells, especially the Foxp3^+^ Treg cells, are critical for maintaining the balance of gut microbiota and the physiological stability of the intestinal tract (Fujimura et al., 2016). The interaction of gut microbiota with intestinal Treg cells requires the regulation of c-MAF (T cell transcription factor), and a deficiency in c-MAF would lead to severe diseases related to the disorder of gut microbiota (Campbell et al., 2018; Xu et al., 2018; Neumann et al., 2019). In addition, the short-chain fatty acids (SCFAs), generated by the gut microbiota, can increase the number of Treg cells in the colon and enhance their ability to secrete IL-10 (Smith et al., 2013; Russler-Germain et al., 2017). IL-22 protects the integrity of the intestinal epithelial barrier and reduces the intestinal permeability of dietary antigens (Brandl et al., 2021). The germ-free mice, colonized with genus *Clostridium*, produced a great amount of IL-22 and reduced the number of allergens entering the bloodstream; the introduction of some *Clostridium* species into gut microbiota alleviated the host allergen sensitization (Figure 1; Stefka et al., 2014). The gut microbiota maintains tolerance to dietary antigens by mediating a protective response to the intestinal epithelial barrier. For example, as a local Ig, IgA protects intestinal mucosa through interacting with gut microbiota and antigenic rejection, thereby participating in immune tolerance (Stefka et al., 2014; Donaldson et al., 2018).

## Microbial Interactions and CMPA

The early-life gut microbiota of infants is associated with the development of the immune system. Several pieces of evidence showed that the development of gut microbiota was associated with the level of IgE, thereby increasing the occurrence of allergy (Sarkar et al., 2021). Therefore, the development of gut microbiota is essential for stimulating the immune system (Cahenzli et al., 2013). The transmission of gut microbiota from mother to infant could help in the development of proper gut microbiota, and the strain-level microbial profiling revealed that the infant’s gut microbiota shifted from maternal vaginal microbiota to distinct maternal sources with selections after birth (Ferretti et al., 2018). The diets have a major impact on the establishment of early-life gut microbiota. The breast-feeding bacteria, such as *Streptococcus* spp. and *Veillonella dispar*, transferred from breast milk to infant gut microbiota, which influenced its development (Di Luccia et al., 2020). Although the gut microbiota of infants is dynamic and variable, it has similar trajectories and different maturation paces (Bäckhed et al., 2015; Weström et al., 2020; Roswall et al., 2021). Moreover, the under-nutrition states affected the response of oral cholera vaccination, suggesting that the diets have global effects on the immune system and might interact with allergy, including CMPA (Di Luccia et al., 2020).

The gut microbiota protects the intestinal barrier and mediates immune tolerance by secreting active metabolites, such as inosine and SCFAs (Mager et al., 2020; Yang et al., 2020), while food provides nutrients and habitat for the microbes (Ma et al., 2017). The gut microbiota of the infants with CMPA was introduced into the germ-free mice, which showed lower sensitivity to the allergic materials of cow’s milk (Feehley et al., 2019), suggesting the potential microbial involvement in the development of CMPA. As compared to that of healthy children, the gut microbiota of the children with CMPA showed enrichment in the relative abundance of families Trichocomaceae and Ruminococcaceae as well as genera *Bacteroides* and *Alistipes* while a decrease in that of genus *Bifidobacterium*, suggesting that the gut microbiota of children with CMPA might be under imbalanced state (Berni Canani et al., 2018; Mauras et al., 2019). A long-term investigation of gut microbiota of children with CMPA showed that the children that recovered from CMPA had enriched Clostridia and Firmicutes, and further metagenomic analyses predicted that their gut microbiota had decreased ability of fatty acid metabolism (Bunyavanich et al., 2016).

## Probiotics, Prebiotics, and CMPA

**FIGURE 2:**
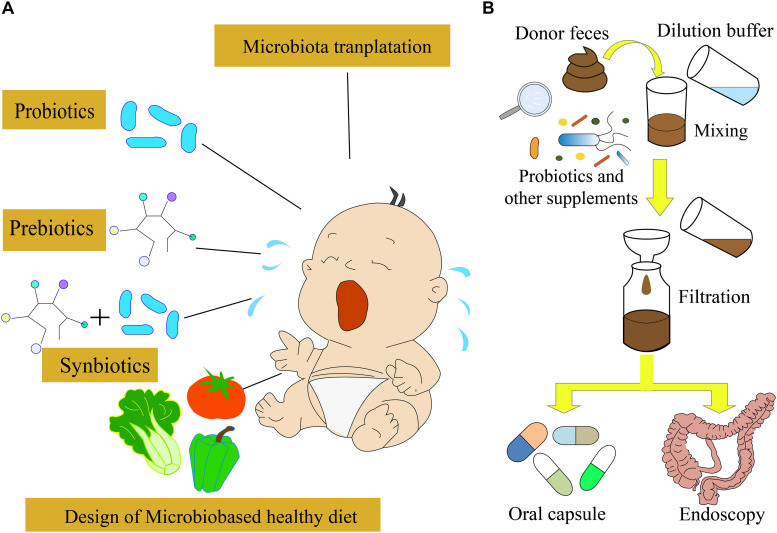
Different strategies for CMPA treatment. **(A)** Probiotics, prebiotics, and synbiotics are often used for CMPA treatment. In the future, the microbiota-targeted design of a healthy diet might be possible to treat CMPA infants. **(B)** Fecal microbiota transplantation strategy used for CMPA treatment.

A low amount of whey was absorbed by the epithelium, most of which was transferred to the Peyer’s patches, suggesting that the physicochemical features of proteins could affect allergic responses (Graversen et al., 2020). In order to alleviate or avoid CMPA, the AAF and deep hydrolyzed milk powder were used as the main food for infants and children with CMPA. Moreover, cow’s milk after heat treatment or being processed by other ways was used for the feeding of infants with CMPA; however, these strategies could not cure CMPA directly (Geiselhart et al., 2021). Avoiding the allergens in food or other materials is difficult in daily life. Therefore, the transfer from food avoidance to active treatment is essential. Omalizumab and oral immunotherapy have been used for treating IgE-mediated food allergy, which has shown the potential to be used for CMPA treatment (Inuo et al., 2018; Costa et al., 2020). Nowadays, a few other active treatment strategies, such as probiotics and prebiotics (Qamer et al., 2019), transplantation of fecal microbiota (Feehley et al., 2019), and precise personalized designed diets, have been developed for the treatment of infants with CMPA (Figure 2).

Probiotics are defined as live microorganisms, which are beneficial for the host’s health. They modulate the structure and function of gut microbiota and interact with the enterocytes through decreasing gut permeability, enhancing mucus thickness, stimulating secretory immunoglobulin A, and producing defensin (Maldonado Galdeano et al., 2019). Moreover, probiotics can modulate the cytokine’s response by immune cells and help in preventing allergies (Berni Canani et al., 2012, 2016; Hardy et al., 2013; Hill et al., 2014; Muraro et al., 2014). Therefore, probiotics and prebiotics have been used for the prevention and treatment of food allergy *via* the modulation of gut microbiota and immune system (Figure 2A; D’Auria et al., 2019). The supplementation of *Lactobacillus* and *Bifidobacterium* species has been used in a practical treatment for CMPA, which accelerated the immune tolerance to cow’s milk in infants with CMPA (Hol et al., 2008; Berni Canani et al., 2012). A randomized, double-blind, and placebo-controlled trial indicated that the probiotics *Lactobacillus rhamnosus* and *Lactobacillus casei* strains could improve the symptoms of infants with CMPA (Cukrowska et al., 2021). A meta-analysis suggested that probiotics could improve the symptoms of CMPA, but there was no evidence of improving tolerance to cow’s milk (Berni Canani et al., 2017; Tan-Lim and Esteban-Ipac, 2018). The tolerance to cow’s milk can be developed if the infants with CMPA can use extensively hydrolyzed formula containing *L. rhamnosus* GG (LGG) (Berni Canani et al., 2012). The LGG is a butyrate producer, which might modulate the expression of genes involved in the allergic pathway, to improve the tolerance to cow’s milk proteins (Berni Canani et al., 2012, 2013; Elce et al., 2017; Nocerino et al., 2019). Moreover, the supplementation of *L. rhamnosus* LA305, *Lactobacillus salivarius* LA307, or *Bifidobacterium longum* subsp. to CMPA-mouse models altered the gut microbiota at the species level and immune responses, which led to the acquisition of tolerance to some food allergies (Esber et al., 2020). However, further assessment of the probiotics supplementation effect on the development of immune tolerance is necessary before the clinical application.

Prebiotics are beneficial substances, which promote the performance of the indigenous microorganisms and host immune system (Hardy et al., 2013; Hill et al., 2014). They are abundant in human breast milk and include galactooligosaccharides (GOS), fructooligosaccharides (FOS), 2′-fucosyllactose, and lacto-*N*-neo-tetraose. They have direct effects on the host by interacting with the host epithelial barrier and indirect effects via the metabolites (Miqdady et al., 2020). They also act as the energy and nutrient sources for selective fermentation by resident health-promoting microorganisms in the gastrointestinal tract, which can protect against pathogens, improve intestinal barrier function, and orchestrate immune pathways (Parnell and Reimer, 2012; Wasilewski et al., 2015).

A mixture of prebiotics could reduce the incidence of allergic responses before 2 years of life and had long-term immune-modulating effects (Arslanoglu et al., 2008). Prebiotics also showed preventive effects in allergy and promoted a tolerogenic environment (Brosseau et al., 2019). The supplementation of prebiotics has been suggested as an effective intervention strategy to treat allergic disorders (Sestito et al., 2020). Although the use of prebiotics has positive effects on the improvement of allergic responses, available evidence is insufficient. Therefore, further global and rigorous studies with randomized, double-blind, and placebo-controlled designs are necessary before the recommendation of any prebiotic as a routine supplementation for the prevention of allergy in formula-fed food. Synbiotics are combinations of useful probiotics and prebiotics, which provide a synergistic effect on human health (Markowiak and Śliżewska, 2017). Compared to probiotics and prebiotics, the design of proper synbiotics would enrich specific taxa in gut microbiota and provide long-term benefits for CMPA infants (Phavichitr et al., 2021).

## Microbiota-Targeted Prevention and Treatment of CMPA

As previously described, the gut microbiota is associated with the occurrence of allergic reactions (Shu et al., 2019). A recent study showed that the gut microbiota of cesarean section-delivered newborns was restored to the normal state similar to the vaginally delivered newborns by maternal fecal microbiota transplantation (FMT) (Korpela et al., 2020), suggesting that FMT can modulate infants’ gut microbiota and might be a possible treatment strategy for CMPA (Albuhairi and Rachid, 2020). FMT is the transfer of normal gut microbiota from healthy people to patients, rebuilding the gut microbiota ecosystem (Figure 2B). It has been applied to treat several diseases, including inflammatory bowel disease (IBD), and *Clostridioides difficile* infection (CDI) (Mohajeri et al., 2018; Galan-Ros et al., 2020; Glassner et al., 2020; Zhang et al., 2020). The survival rate of IBD caused by CDI using the FMT strategy could reach 90% (Basson et al., 2020). The animal trials demonstrated that FMT could improve CMPA symptoms. However, the use of FMT for the CMPA treatment is still under development. As the gut microbiota of infants is immature (Stokholm et al., 2018; Moore and Townsend, 2019), the curative effects of FMT on CMPA treatment need comprehensive evaluation.

Nowadays, the incidences of food allergy have been increased due to the alterations in genome–environment interaction and modern lifestyles (Loh and Tang, 2018). Diets play vital roles in food allergy and the development of CMPA, and the early dietary interventions have been proved to be an effective strategy to prevent food allergy (Du Toit et al., 2016). The introduction of solid food at the early life might reduce the incidence of food allergy (Caffarelli et al., 2018; Mastrorilli et al., 2020). High-fiber foods lead to the high level of SCFAs’ release, which might enhance oral tolerance and protect the host from food allergy (Tan et al., 2016; Makki et al., 2018). In contrast, high-fat diet induces post-diet alteration in gut microbiota, which might increase the incidence of food allergy (Hussain et al., 2019). Moreover, the extensive casein formula, supplemented with LGG and EHCF which designed for the treatment of infants with CMPA, significantly increased the fecal butyrate levels, which increased the infants’ tolerance to CMPA (Berni Canani et al., 2016) via the alteration of gut microbiota (Berni Canani et al., 2016).

A recent study showed that cranberries attenuated the impact of an animal-based diet to a less favorable profile (Rodríguez-Morató et al., 2018), suggesting that healthy food can induce changes in the composition and function of human gut microbiota. The baseline gut microbiota could affect the final diet intervention results based on a diet-induced weight-loss study, and the abundance of some microbial species, including *Ruminococcus gnavus*, *Akkermansia muciniphila*, *Blautia wexlerae*, and *Bacteroides dorei*, was found to be linked to the weight loss during diet interventions (Jie et al., 2021). Moreover, the changes in gut microbiota induced by the diet were temporary and the long-term dietary interventions are still unknown (Leeming et al., 2019). Therefore, the modulation of gut microbiota should consider the personal baseline microbiota and personalized responses to diets. The microbiota-based design of healthy food is crucial for the personalized nutrient supplementation strategy in the future (Fan and Pedersen, 2021). After understanding the keystone microbial taxa in infants with CMPA and diet–microbiota interactions, the design of personalized diets might contribute to the CMPA treatments (Figure 2A; Kolodziejczyk et al., 2019). With the development of synthetic biology and computational approaches, the intervention of engineered live microbiota that produces active molecules into infants with CMPA is a potential treatment strategy for CMPA via the integration of multi-omics data and clinical characteristics (Guan et al., 2020; Wang et al., 2020; Peng et al., 2021).

## Future Perspectives and Conclusion

An insight into the gut microbiota of infants with CMPA and the identification of keystone taxa in its development are required for the diagnosis and treatment of CMPA. Moreover, current focus should be shifted from the descriptive CMPA gut microbiota to the cause-and-effect host–microbiota investigation, which will reveal the CMPA-related microbiota. The global profiling of long-term changes and the dietary intervention effects on gut microbiota are required for the dietary modulation of CMPA gut microbiota. Besides, the supplementation of probiotics, prebiotics, synbiotics, FMT, and microbiota-based design of a healthy diet is intrigued to implement for the treatment of CMPA in the future. Based on the relationship of gut microbiota and CMPA, the microorganism-based diagnosis and treatment strategies of CMPA might be developed soon, which may improve the health and life quality of CMPA infants.

## Author Contributions

YW and BJ conceived the study. YDY, XL, and YY drafted the manuscript. YDY prepared the figures. CZ and SS revised the manuscript. YW and BJ designed the whole study and revised the manuscript. All authors read, revised, and approved the manuscript.

## Conflict of Interest

The authors declare that the research was conducted in the absence of any commercial or financial relationships that could be construed as a potential conflict of interest.

## Publisher’s Note

All claims expressed in this article are solely those of the authors and do not necessarily represent those of their affiliated organizations, or those of the publisher, the editors and the reviewers. Any product that may be evaluated in this article, or claim that may be made by its manufacturer, is not guaranteed or endorsed by the publisher.
