# Supplementary material for: Advances in the Relationships Between Cow’s Milk Protein Allergy and Gut Microbiota in Infants
Source: Front Microbiol. 2021 Aug 16;12:716667. doi: 10.3389/fmicb.2021.716667 (PMC8415629; doi:10.3389/fmicb.2021.716667)
Supplement: Supplementary file 2 [file Image_1.pdf]

Advances in the relationships between Cow's milk protein allergy and gut microbiota

Yudie Yang[1], Xiaoqi Li[1], Ying Yang[4], Saeed shoaie[5,6], Cheng Zhang[1,5], Boyang Ji[2*], Yongjun Wei[1,3*]

[1] Key Laboratory of Advanced Drug Preparation Technologies, Ministry of Education, School of Pharmaceutical Sciences, Henan Children's Hospital, Zhengzhou Children's Hospital, Children's Hospital Affiliated to Zhengzhou University, Zhengzhou University, Zhengzhou 450001, PR China

[2] Jing'an District Central Hospital of Shanghai, Jing'an Branch, Huashan Hospital, Fudan University, 220 Handan Road, Shanghai, 200433, PR China

[3] Science for Life Laboratory, KTH - Royal Institute of Technology, SE-171 21, Stockholm, Sweden

[4] Centre for Host-Microbiome Interactions, Faculty of Dentistry, Oral & Craniofacial Sciences, King's College London, SE1 9RT, UK

[5] Department of Biology and Biological Engineering, Chalmers University of Technology, Gothenburg, Sweden

[6] Laboratory of Synthetic Biology, Zhengzhou University, Zhengzhou 450001, PR China

*Corresponding author

Yongjun Wei

E-mail: yongjunwei@zzu.edu.cn

Boyang Ji

E-mail: boyang.ji@chalmers.se

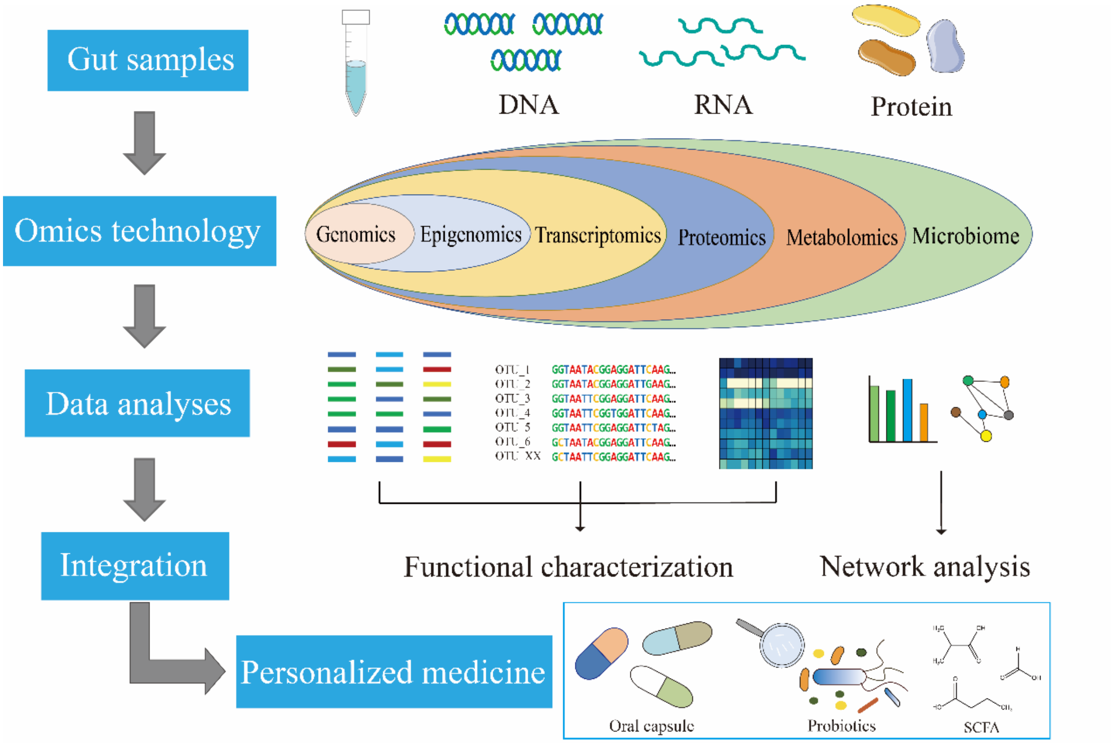

Figure S1 The omics technologies used to give insights into the interactions between gut microbiota and CMPA. Colleting gut samples, and using omics technology can give insight into the interactions between omics data and other physiological characteristics of CMPA infants. Based on the data of functional characteristics and other microbial networks, the biomarkers for CMPA can be revealed, and personalized medicine can be designed to treat of CMPA.
